# Psychosocial disadvantage and residential remoteness is associated with Aboriginal women’s mental health prior to childbirth

**DOI:** 10.23889/ijpds.v5i1.1153

**Published:** 2020-02-26

**Authors:** SK Bhat, R Marriott, M Galbally, CCJ Shepherd

**Affiliations:** 1 Ngangk Yira: Murdoch University Research Centre for Aboriginal Health and Social Equity, Australia; 2 School of Psychology and Exercise Science, Murdoch University, Australia; 3 School of Medicine, University of Notre Dame, Australia; 4 King Edward Memorial Hospital, Australia; 5 Telethon Kids Institute, The University of Western Australia, Nedlands, Australia

## Abstract

**Introduction:**

Optimal mental health in the pre-conception, pregnancy and postpartum periods is important for both maternal and infant wellbeing. Few studies, however, have focused on Indigenous women and the specific risk and protective factors that may prompt vulnerability to perinatal mental disorders in this culturally diverse population.

**Objectives:**

To assess mental health contacts in the period before childbirth among Australian Aboriginal and Torres Strait Islander women, the association with socioeconomic factors and whether it differs by geographic remoteness.

**Methods:**

This is a retrospective cohort study of 19,165 Aboriginal mothers and includes all Aboriginal mothers and their children born in Western Australia from January 1990 to March 2015. It draws on population-level, linked administrative data from hospitals and mental health services, with a primary focus on the mental health contacts of Aboriginal women in the 5 years leading up to childbirth.

**Results:**

The prevalence of maternal mental health contacts in the five years prior to birth was 27.6% (93.6% having a single mental health disorder), with a greater likelihood of contact in metropolitan areas compared with regional and remote settings. There was a positive relationship between socioeconomic advantage and the likelihood of a mental health contact for women in Metropolitan (β = 0.044, p=0.003) and Inner regional areas (β = 0.033, p=0.018), and a negative association in Outer regional (β = -0.038, p=0.022), Remote (β = -0.019, p=0.241) and Very remote regions (β = -0.053, p<0.001).

**Conclusions:**

The findings from this study provide new insights on the dynamic relationship between SES, geographic location and mental health issues among Aboriginal women in the 5 years leading up to childbirth. The results underscore the need to apply location-specific approaches to addressing the material and psychosocial pathways that lead to mental health problems and the provision of culturally safe, appropriate and accessible services for Aboriginal women

## Introduction

The existing literature clearly supports the importance of maternal mental health from pre-conception, through pregnancy and the postpartum for both maternal and infant wellbeing[1,2]. This includes the direct impact of maternal mental health on both parenting and intergenerational mental health and developmental outcomes for children[3]. However, much of what is known is based on research among urban women and with limited cultural diversity in many studies. In particular, there is very limited research on Indigenous women to understand the specific risk and protective factors that may specifically underlie their vulnerability to perinatal mental disorders. This raises significant questions about the gaps in our current understanding and capacity to develop clear guidelines and recommendations for assessment and management that are both culturally appropriate and evidence based for women within Indigenous communities[4].

Within Australia, the qualitative and ethnographic literature clearly highlight there is poorer mental health among Indigenous Australians—including Aboriginal and Torres Strait Islander peoples (hereafter referred to as *Aboriginal*)—than in non-Aboriginal Australians[5,6]. This evidence is supported by a limited quantitative evidence base[7,8], though few provide an insight into maternal wellbeing and related inequalities during pregnancy and the postpartum. This, in part, reflects the difficulties in measuring and assessing mental health in culturally distinct populations[9-11]. A broad range of factors have been shown to influence the development of mental health problems, including the social[12], economic and psychological conditions of families[13]. In addition, it is now well accepted that the post-colonial history of Aboriginal Australia, characterised by discriminatory policy and practices, widespread dispossession and exclusion, has been particularly harmful to the social and emotional wellbeing of Aboriginal peoples and served to perpetuate a burden of disadvantage across generations of Aboriginal families[14,15]. Despite a large sum of money [$9.1 billion (Australian dollars)] spent on mental health in Australia during year 2016–17 the rate of community mental health care utilisation by Aboriginal patients is more than three times that of non-Aboriginal patients, with young females aged 12–17 years having the highest community mental health care service contact rate in 2017–18[16].

The contribution of socioeconomic status (SES) to increasing vulnerability to mental health problems is a feature of the quantitative empirical literature, with studies typically showing better socioeconomic outcomes being associated with better mental wellbeing[13,17]. While this pattern is also evident in Aboriginal populations[13,18-20], we have a limited understanding of how both socioeconomic disadvantage and residential remoteness may impact perinatal mental health in Aboriginal women specifically [21-26]. Within Australia, the impact of location and geographic remoteness is important in understanding Aboriginal women’s mental health given the unique geographic dispersion of Aboriginal populations. Furthermore, our understanding of the impact of regional, rural and remote residence on perinatal mental health for women living in these communities is extremely limited. Within the existing literature there is little consideration of the context or differences between rural communities, with even less understanding of Aboriginal women residing in rural or remote communities [16].

There are a number of potential differences for Aboriginal women living in rural and remote communities across the perinatal period and this includes geographical distances associated with isolation and access to specialist services, health promoting lifestyle factors (e.g. diet, exercise, smoking, sleep and alcohol use), social support, stressful life events (including violence and financial issues) and the impact of natural environment that have all been shown to differ for those living in rural communities[8,27,28]. Stress, for example, has been reported as a more prevalent phenomenon among Aboriginal peoples in remote areas; commonly precipitated by the death of a family member or close friend, overcrowding at home, alcohol or drug-related problems, serious illness, disability, having a family member sent to jail and family financial strain[6,20,29]. Equally, residing in communities that are culturally safe with connection to family, community and social support is likely to be protective for mental health even when this is associated with geographical distance[7].

There is some empirical evidence of the separate effects of SES and remoteness on mental health contacts among Aboriginal women, but the combined effect is not well understood. Australian studies assessing the association between remoteness and mortality or other health outcomes have consistently found worse outcomes for those living outside of major cities[30,31]. Remoteness has an effect above and beyond SES for a number of subpopulations for cardiovascular mortality[31]. Western Australia (WA), a state of Australia, is an ideal setting to examine the impact of residence and remoteness on perinatal mental health of Aboriginal women, with significant environmental and culture diversity across a vast geographic landscape. An improved understanding of both the geographical and social factors associated with Aboriginal women’s mental health across the childbearing years is important if we are to develop recommendations that support better mental health outcomes for women, their children, families and communities. This, in turn, can also provide more nuanced guidance to population and public health policies aimed at reducing mental health prevalence.

This study makes use of unique, population-level linked administrative data from hospitals and mental health clinics across WA, with a primary focus on the mental health contacts for Aboriginal women in the 5 years leading up to childbirth. Specifically, we aimed to: (1) assess whether there is a social gradient in Aboriginal women’s mental health contacts in the 5 years prior to childbirth; and (2) determine if the socioeconomic pattern of mental health contacts differs by geographic remoteness. A better understanding of the combined effect will help us to disentangle the relationship between issues of access and the enabling resources of relative socioeconomic advantage.

## Methods

### Data source

This study used de-identified linked administrative data from the WA Department of Health that included the Hospital Morbidity Data Collection (HMDC), Mental Health Information System (MHIS), Midwifes Notification System (MNS), and Birth Register (BR). The HMDC contains detailed information on all inpatient episodes in public and private hospitals in WA (1970-current), with admissions coded according to the International Classification of Diseases (ICD). The MHIS contains information on all mental health-related public and private inpatient admissions and public outpatient contacts (1966–current). The MNS records the circumstances of all births of 20 weeks of gestation or more, with information received from attending practitioners since 1980 and was used as the primary data source to establish the cohort. The Birth Register dataset has information on all births registered in WA (1974-current), containing information from the mother, father and baby. The datasets have been shown to be highly reliable[32,33] and were linked by the WA Data Linkage Branch using probabilistic matching and a robust and internationally accepted privacy preserving protocol[34]. Only de-identified data were provided to the research team, via secure transfer.

### Study design

This is a retrospective cohort study that includes all Aboriginal women and their children born in Western Australia from January 1990 to March 2015. Births were identified from the MNS and Birth Registrations. Indigenous status was derived using the *Getting Our Story Right* indicator, developed by Christensen et al[35] using a multi-stage median approach across a wide range of datasets to derive an Indigenous status for each study child. The indicator is considered an optimal approach to identifying Aboriginality in administrative datasets[35].

### Primary outcome– Maternal mental health contacts

The mental health system in WA includes a mix of public and private services provided in a range of settings—hospitals, community mental health units or centres, and private practitioners (GPs) or other mental health specialists. In general, patients are referred from a GP or other health providers when mental health services are required. Patients can receive mental healthcare as an inpatient — admitted to hospital, clinic or other mental health service — or as an outpatient, when they receive treatment without being admitted to hospital. Aboriginal Australians can access mainstream or Aboriginal-specific services, with Aboriginal-specific services generally available through community clinics, services provided by Aboriginal Community Controlled Health Organisations and other healthcare facilities, and some public hospitals. Maternal mental health contacts were identified using mental-health-related inpatient hospital admissions (from HMDC) and mental-health-related outpatient contacts (from MHIS) and classified using ICD-10-AM codes (International Statistical Classification of Diseases and Related Health Problems-Tenth edition-Australian Modification). Mapping tables were used to recode different editions of ICD codes. Mothers were classified as having a mental health-related admission or contact if they had a mental health-related ICD diagnosis (primary and/or secondary).

For the purposes of this study, we coded all maternal mental health contacts that occurred in the five years prior to birth, with each contact coded to one of ten mental health condition categories, based on ICD-10-AM (Supplementary table 1). The ten conditions were Substance-related disorder, Schizophrenia, Mood disorder, Anxiety, Personality disorder, Intellectual disability (IQ), Disorders of psychological development, Intentional self-harm, Organic mental health disorder and Other. The ‘other’ mental health related diagnosis group included contacts for which the principal or secondary diagnosis was relevant to mental health but did not meet the specific criteria for a mental health diagnosis. These consisted of contacts and issues that are generally considered less severe than those of diagnoses-specific groups.

### Primary exposure– Geographic regions and remoteness

We included two geographic variables in order to capture aspects of remoteness and the characteristics of contiguous geographic regions—both form part of the Australian Bureau of Statistics’ (ABS) Australian Statistical Geography Standard. Geographic remoteness was defined according to the 2011 Remoteness Areas (RAs) classification. The classification divides Australia into broad regions that reflect differences in access to services, cultures and health outcomes for Aboriginal women and children. The RAs are based on the Accessibility/Remoteness Index of Australia (ARIA+), and include five categories—ranging from a major city (Perth Metropolitan area), to Inner regional, Outer regional, Remote and Very remote areas[36,37]. We had access to RA data calculated using the Australian Census of Population and Housing in 1996, 2001, 2006 and 2011, and aggregating to Statistical Local Areas (SLA). RA values were assigned based on year of birth of the child in the MNS—for example, a child born during 1990-2000 was assigned the relevant RA value for their mother’s SLA of usual residence in 1996. In addition, we used administrative health service region boundaries to divide WA into ten contiguous geographic regions. These included North, South and East Metropolitan regions within Perth, the capital city of WA; the Goldfields, Southwest, Kimberley, Pilbara, Midwest, Great Southern and Wheatbelt regions. Each of these regions outside of Perth has at least one regional town centre. WA is Australia’s largest state and one of the most ecologically diverse.

### Primary exposure– Index of Relative Socio-Economic Advantage and Disadvantage 

The Socio-Economic Indexes for Areas (SEIFA) product, developed by the ABS, was used to measure area-level socioeconomic status. SEIFA ranks the relative level of disadvantage of areas using the attributes of all persons (Aboriginal and non-Aboriginal) in small areas, and includes measures of income, educational attainment, employment status and occupational skill[38]. For the purposes of this study, we have categorised the SEIFA Index of Relative Advantage/Disadvantage (IRSAD) into deciles, based on 2006 Census values for children born from 1990-2010, and 2011 Census values for all other participants.

### Covariates

Maternal age (single years) and maternal marital status at the time of a childbirth were recorded in the Birth Registration dataset. Parity is recorded as a discrete variable for each birth in the MNS.

### Statistical analyses

Data analyses were performed using Stata 13 (StataCorp, College Station, TX, USA). Summary estimates for continuous and categorical variables were calculated as means or proportions with 95% confidence intervals (CIs). Models were run on the sample of Aboriginal children born between January 1990 and March 2015, inclusive. A series of regression models were used to assess the relationship between maternal mental health contacts, area-level socioeconomic status (IRSAD-decile score) and geographic remoteness. First, log-odds regression models regressed the maternal mental health contacts log-odds/probability on geographic remoteness and IRSAD, without an interaction-term. This unadjusted analysis provided the respective marginal estimates for remoteness and IRSAD with no statistical interaction. Covariates were subsequently added to the unadjusted model in a step-wise fashion to achieve the fully-adjusted log-odds regression model (marital status, followed by maternal age and maternal parity). Akaike’s Information Criterion (AIC) and Bayesian Information Criterion (BIC) were used to compare model fit, ranging from basic to multivariable log-odds models—with lower values indicating a better model fit. Next, we compared the results of the mixed effects log-odds regression model (with no interaction-term) with the last multivariable log-odds regression model. Likelihood ratio (LR) test versus logistic regression chi-squared statistic (with one degree of freedom) determined goodness of fit for the mixed effects model. Log-odds regression models are presented as Models 1-3 whereas Model 4 is a mixed effects log-odds regression, allowing random intercepts for maternal health service utilisation. The final mixed effects log-odds regression model incorporated an interaction-term between IRSAD and remoteness. The two nested mixed effects models, with and without interaction-term, were compared with the nested LR ratio. The final mixed effects log-odds regression model (with the interaction-term) was applied to all Aboriginal children born between January 1990 and March 2015, thereby including multiple records per mother and allowing for multiple health service contacts per mother across all births in the study period.

## Results

The final study sample included 19,165 mothers and their 39,845 Aboriginal children born between January 1990 and March 2015 and represents the participants with complete information on primary predictors (IRSAD decile score, geographical remoteness) and other covariates (88.1% of all 45,211 in-scope children). The prevalence of maternal mental health contacts in the five years prior to birth was 27.6% (n=10,978), with the majority (93.6%) of these having a single mental health disorder.

The proportion with a maternal mental health contact was similar between the study sample and those excluded. However, our sample was more commonly from lower SES areas and Inner regional and Very remote settings and had a younger maternal age. The mothers of almost two-thirds (63.8%) of our sample were married compared with 36.7% of mothers of the excluded sample (Supplementary table 2).

**Table 1: Prevalence of maternal mental health contacts in the 5 years prior to birth, by selected characteristics:** IRSAD = Index of Relative Socioeconomic Advantage and Disadvantage

Independent variables	Observation (n)	%	[95%CI]
Overall Mean IRSAD	10978	5.4	[5.4,5.5]
	
IRSAD deciles 1-2	2873	26.2	[25.3,27.0]
IRSAD deciles 3-4	1743	15.9	[15.2,16.6]
IRSAD deciles 5-6	1280	11.7	[11.1,12.3]
IRSAD deciles 7-8	2549	23.2	[22.4,24.0]
IRSAD deciles 9-10	2533	23.1	[22.3,23.9]
Remoteness Areas	10978	
	
Metropolitan	2816	25.6	[24.8,26.5]
Inner regional	1981	18.0	[17.3,18.8]
Outer regional	1056	9.6	[9.1,10.2]
Remote	837	7.6	[7.1,8.1]
Very remote	4288	39.1	[38.1,40.0]
Maternal marital status	10978	
	
Not married	3950	36.0	[35.1,36.9]
Widowed	18	0.2	[0.1,0.2]
Divorced	27	0.2	[0.2,0.3]
Separated	278	2.5	[2.2,2.8]
Married/de facto	6560	59.8	[58.8,60.7]
Not stated	145	1.3	[1.1,1.5]
Overall Mean Maternal age	10978	25.4	[25.3,25.5]
	
<24 years	5055	46.1	[45.1,47.0]
25-34 years	4904	44.7	[43.7,45.6]
≥35 years	1019	9.3	[8.7,9.8]
Maternal parity	10978	
	
Parity: none	2507	22.8	[22.1,23.6]
Parity: 1+	8471	77.2	[76.4,77.9]

More than half of the maternal mental health care service contacts were in Outer regional, Remote and Very remote areas (56.3%) closely followed by Metropolitan and Inner regional areas (43.6%) (Table 1). Mean age of 25.4 years for mental health contacts suggested a sample of young mothers though nearly 10% of mental health contacts were those with an advanced age of 35 or more years.. More than two-thirds (77.2%) maternal health contacts had at least one child and 59.8% contacts were married (Table 1).

Supplementary figure 1 showing the influence of IRSAD and remoteness covariate patterns illustrates the change in Pearson chi-squared by predicted margins (probability) of mental health contact to be appropriate, with no significant outliers. Supplementary table 3 shows results for the adequacy of model fit comparing model fit statistics, Akaike’s Information criterion (AIC) and Bayesian information criterion (BIC), across all the models, including the mixed effects models. The lower values of AIC and BIC indicated a better model fit. Table 2 - Model 4 mixed effects model with no interaction (Supplementary figure 2) was not superior to the mixed effects model with the interaction-term between SES and remoteness (Table 3).

Table 2- Model 4 mixed effects model allowed random intercept for health service regions, thereby adjusting for variability in the contacts across health service regions. The random effects parameter estimate indicated significant variation in maternal mental health contacts by region, the standard deviation of random intercepts being nearly four standard errors from zero (0.178) (Table 2 footnote). The LR test for mixed effects Logistic regression (Model 4) versus fixed effects Logistic regression (Model 3) was significant (Table 2 footnote).

**Table 2: Log-odds regression models predicting probability of maternal mental health by IRSAD and geographic remoteness:** 95% confidence intervals in brackets * p < 0.05, ** p < 0.01, *** p < 0.001 ^a^ Remoteness Areas and socioeconomic IRSAD decile score ^b^ Maternal marital status ^c^ Maternal age and maternal parity ^d^ Likelihood Ratio test for mixed effects (Model 4) versus Logistic regression (Model 3): chibar2(01) = 112.34 Prob>=chibar2 = 0.0000. ^¥^ Random-effects Parameter Estimate for Health regions [sd(cons)=0.178 Sth. Err.(0.046); 95% CI: 0.107, 0.297]

	Unadjusted log-odds model (Model 1)a	Partially adjusted log-odds model (Model 2)b	Fully adjusted log-odds model (Model 3)c	Mixed effects log-odds model (Model 4)d
Remoteness Areas				
Metropolitan (Baseline)				
Inner regional	-0.254***	-0.228***	-0.238***	-0.196***
	[-0.321,-0.186]	[-0.296,-0.160]	[-0.307,-0.169]	[-0.267,-0.126]
Outer-regional	-0.273***	-0.237***	-0.219***	-0.188***
	[-0.360,-0.185]	[-0.325,-0.149]	[-0.309,-0.129]	[-0.283,-0.093]
Remote	-0.512***	-0.485***	-0.467***	-0.472***
	[-0.606,-0.418]	[-0.580,-0.391]	[-0.564,-0.371]	[-0.575,-0.370]
Very remote	-0.397***	-0.351***	-0.335***	-0.393***
	[-0.467,-0.327]	[-0.422,-0.280]	[-0.407,-0.263]	[-0.479,-0.306]
IRSAD-decile score	-0.038***	-0.038***	-0.036***	-0.019***
	[-0.048,-0.029]	[-0.048,-0.029]	[-0.046,-0.027]	[-0.030,-0.008]
Marital Status				
Not married (Baseline)				
Widowed		0.355	0.127	0.117
		[-0.229,0.938]	[-0.460,0.714]	[-0.472,0.706]
Divorced		0.06	-0.263	-0.269
		[-0.395,0.516]	[-0.728,0.201]	[-0.735,0.197]
Separated		0.526***	0.242**	0.228**
		[0.368,0.685]	[0.080,0.404]	[0.066,0.391]
Married/de facto		-0.157***	-0.317***	-0.319***
		[-0.204,-0.111]	[-0.367,-0.267]	[-0.369,-0.269]
Unknown		0.207*	0.143	0.151
		[0.005,0.410]	[-0.063,0.349]	[-0.057,0.358]
Maternal age at tde time of childbirtd			0.013***	0.013***
			[0.008,0.018]	[0.008,0.018]
Maternal parity (one or more children)			0.109***	0.108***
			[0.093,0.124]	[0.092,0.124]

Observations	40794	40794	39869	39845

Random-effects Parameters for Health regions	Not applicable	Not applicable	Not applicable	Applicable ¥

**Figure 1: Random intercept estimates for maternal mental health contacts, by health service regions of Western Australia:**
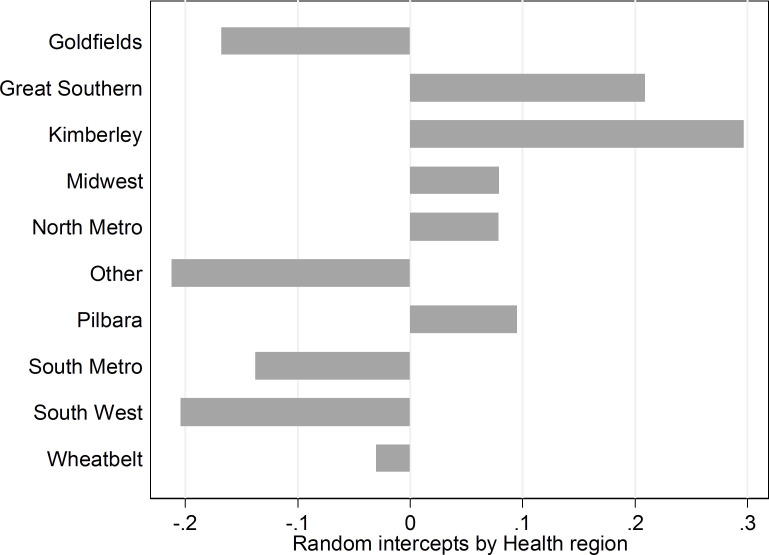


Figure 1 illustrates distinct regional differences in maternal mental health contacts, after allowing for socioeconomic status, geographic remoteness, marital status, maternal age and parity. Large positive random y-intercepts were observed for Great Southern, Kimberley, Pilbara, Midwest, and North Metropolitan regions, with negative random y-intercepts for the South West, Goldfields, and Wheatbelt regions and the South Metropolitan area.

**Figure 2: Predicted margins (log-odds) of mental health contact by geographic remoteness and IRSAD interaction:**
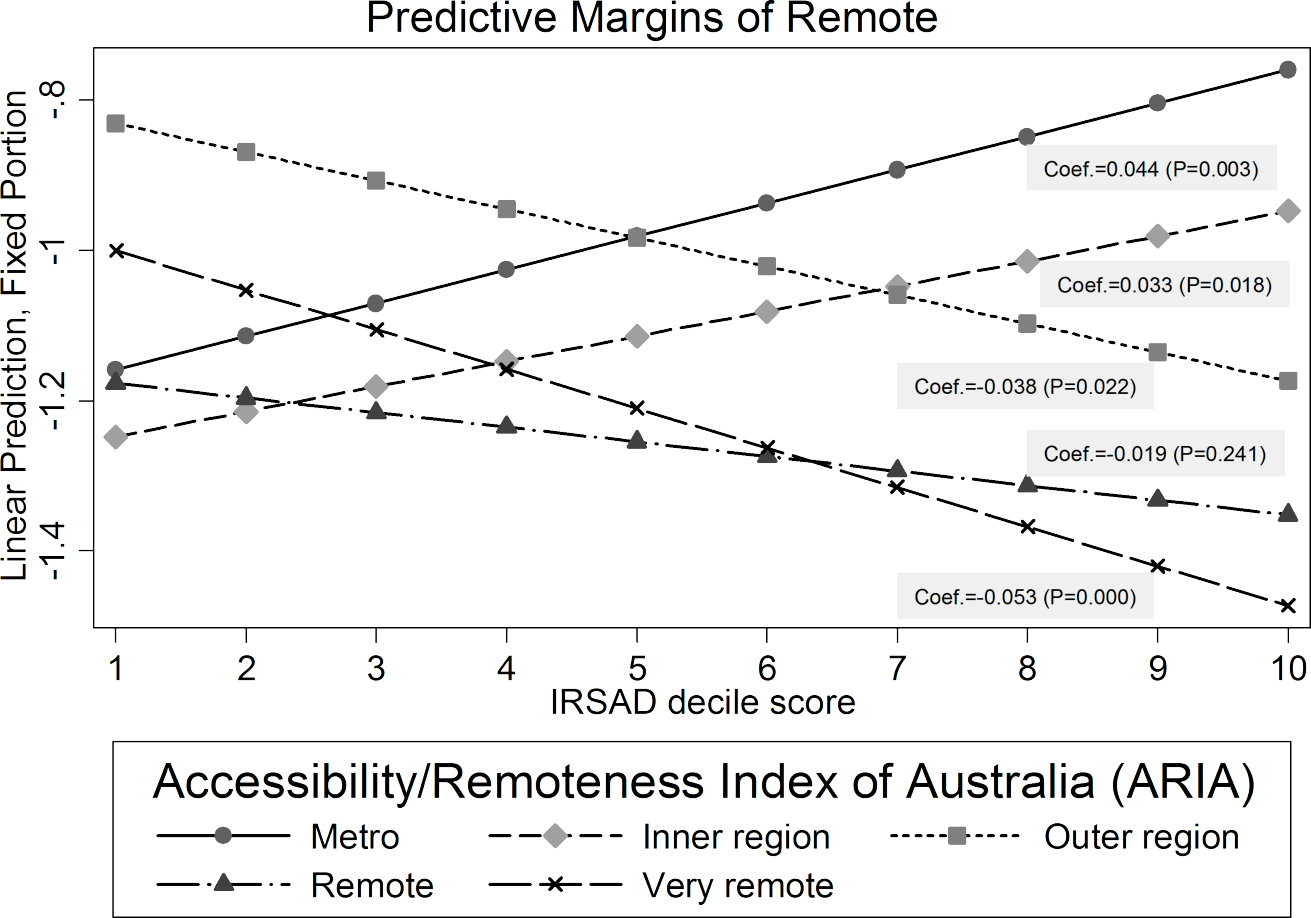


When specifying an interaction between IRSAD and remoteness, we observed differences in the fixed effect interaction-term coefficients (Table 3, Figure 2, and Supplementary figure 3). The mixed effects log-odds model estimates highlight statistically significant effects for the interaction terms for Outer regional (-0.038 minus 0.044 equal to -0.082), Remote (-0.019 minus 0.044 equal to -0.064), and Very remote (-0.053 minus 0.044 equal to -0.097) areas when compared with the Metropolitan area. In other words, the effect of SES (IRSAD decile slope) for Metropolitan versus Outer regional, Metropolitan versus Remote, and Metropolitan versus Very remote was 7.9%, 6.2%, and 9.2% higher, respectively.

**Table 3: Mixed effects multivariable logit regression model predicting log-odds of maternal mental health contacts by IRSAD-decile score and geographic remoteness interaction:** 95% confidence intervals in brackets * p < 0.05 ** p < 0.01 *** p < 0.001

Observations=39,845	Final mixed effects logit-model with interaction term	

	Coefficient	95% Conf. Interval

Metropolitan (Baseline)
Inner regional	-0.078	[-0.395,0.239]
Outer-regional	0.410**	[0.115,0.706]
Remote	0.046	[-0.245,0.337]
Very remote	0.256*	[0.002,0.509]
IRSAD decile score	0.044**	[0.015,0.073]
Interaction between Metropolitan and IRSAD (Baseline)
Interaction between Inner regional and IRSAD	-0.011	[-0.051,0.029]
Interaction between Outer regional and IRSAD	-0.082***	[-0.126,-0.039]
Interaction between Remote area and IRSAD	-0.064**	[-0.107,-0.020]
Interaction between Very remote area and IRSAD	-0.097***	[-0.130,-0.064]
Not married (Baseline)
Widowed	0.116	[-0.474,0.706]
Divorced	-0.273	[-0.739,0.193]
Separated	0.224**	[0.062,0.387]
Married/de facto	-0.315***	[-0.365,-0.265]
Not stated	0.155	[-0.053,0.362]
Maternal age at the time of childbirth	0.013***	[0.008,0.018]
Maternal Parity	0.109***	[0.093,0.124]
Model constant (_cons)	-1.527***	[-1.806,-1.247]

Random-effects Parameters for Health regions	[sd(cons)=0.159 Std. Err.(0.043)	[0.094,0.271]

Other significant pairwise comparisons of marginal predictions were observed between Remote and Inner regional (Coefficient=-0.053, p=0.016), Very Remote and Inner regional (Coefficient=-0.086, p=0.000), and Outer regional and Inner regional areas (interaction coefficient=-0.071, p=0.001). All the pairwise comparisons involving Very Remote, Remote and Outer regional were not statistically significant (Supplementary table 4). The coefficients for Remote versus Metropolitan areas (-0.064, p=0.083) and Remote versus the Inner regional areas (-0.053, p=0.210) were attenuated after adjustment for multiple comparisons (Supplementary table 5).

## Discussion

Our study found that, among Aboriginal women, higher socioeconomic status (SES) was associated with a decreased likelihood of a contact with mental health services in the five years prior to childbirth. This is consistent with the broader empirical literature, which has demonstrated that socioeconomic advantage is associated with lower risk of mental illness in both child and adult populations[12,39]. While this pattern is also evident in the small number of studies conducted among Aboriginal Australians[13], none have focused on the perinatal period. Our results specifically indicate that neighbourhood-level disadvantage has a strong effect on mental health contacts of Aboriginal mothers in the period prior to childbirth. While this may be a reflection of the mechanisms by which SES impacts the development and manifestation of mental health problems (including direct and indirect material and psychosocial pathways), it is also likely to reflect the pragmatic limitations of living in poorer neighbourhoods with fewer resources for accessing specialist mental health care.

Our study provides new insights on the dynamic relationship between SES and geographic location, highlighting that the pattern of socioeconomic disparities in maternal mental health contacts varied by remoteness of residence. A reverse gradient was observed in urban settings (Metropolitan and Inner regional areas), whereby mental health contacts were more likely to occur in mothers living in less disadvantaged areas. While those living in urban areas typically have access to a broader range of mental health services [40,41], Aboriginal Australians face a range of barriers to services that extend beyond proximity, to issues that are intrinsically linked with disadvantage. These include affordability and the perceived cultural safety, security and competence of service providers and practitioners. Cultural barriers stem from a historical mistrust of mainstream services by Aboriginal peoples [42], which has been fuelled by experiences that were commonly characterised by discrimination and prejudice and the broader role of Australian Government and non-government institutions in past policies and practices that lead to the forced separation of Aboriginal people in Australia from family and kinship networks[14,43].

In contrast, the findings in more remote areas are consistent with a conventional social patterning of health model. This may reflect the myriad pathways by which SES shapes mental wellbeing or the distribution of mental health-related services in more remote settings. While Aboriginal-specific services—including community clinics and services provided by Aboriginal Community Controlled Health Organisations (ACCHOs)—provide an important first point of contact for many Aboriginal people, there are significant gaps in the provision of these services in Remote and Very remote parts of WA[44]. As a result, this may lead more disadvantaged Aboriginal people in these areas to seek support preferentially from hospital settings, instead of specialist and other higher cost options.

The study affirms that mental health issues place a considerable burden on Aboriginal mothers and families in the perinatal period, with over a quarter of Aboriginal mothers having a mental health contact in the five years prior to birth. While our results further implicate socioeconomic status as an important, distal determinant of mental health disorders and problems, it is part of a complex etiology[17]. In the context of Aboriginal Australia, this etiology includes discrimination, stress and the cumulative, inter-generational and pervasive legacies of a colonial history[45]—all of which have had a traumatic negative effect on the social and emotional wellbeing of Aboriginal people over time[46]. Stress, in particular, has been shown to be highly prevalent in Aboriginal populations[6,20,41,47], with a considerable literature demonstrating the adverse impact of intrauterine and antenatal exposure to stress on pregnancy health and the health of children at birth and into childhood[48,49]. In addition to the direct effects of stress and discrimination, these factors can also indirectly impact mental wellbeing by creating barriers to optimal health care and the range of resources that are required to support family planning and a healthy pregnancy[50].

We found distinct regional differences in maternal mental health contacts, even after adjusting for geographic remoteness and socio-demographic factors—whereby the average level of contacts was particularly high in the Kimberley and Great Southern regions, and lower in the South West, Goldfields, South Metropolitan and Other areas (including the Central and East Metropolitan areas). This is likely to reflect the distribution of mainstream and Aboriginal-specific services—including the location of hospitals, clinics and other mental health services. The Kimberley, for example, has a high level of specialist mental health services[51], which are provided in a range of regional centres and via mobile outreach services to smaller and more remote locations. ACCHOs are key providers of these services, and typically provide holistic, culturally-centred services—which can alleviate some of the impediments to seeking care and lead to access levels that corresponds more loosely to the burden of mental health problems in Aboriginal populations[52].

Collectively, the study results underscore the need for location-specific approaches to addressing the material and psychosocial pathways that lead to mental health problems among Aboriginal populations, including those that manifest in the period of pregnancy and early motherhood. The Looking Forward project has reinforced the notion that local context is critical in the design of effective mental health services for Aboriginal peoples[53]. It featured a co-design model, with an emphasis on local community Elder and member input and an Aboriginal worldview as central to the process of developing improved models of mental health care tailored to their community—the Nyoongar community in Perth, WA. This approach is predicated on the notion that culturally-safe specialist mental health services are more likely to be accepted by, and fit the needs of, local community members, and lead to earlier intervention and a greater likelihood of preventing poor downstream consequences of mental illness.

In the context of perinatal and pregnancy health, previous research has highlighted the need for policies directed at the development of a multisector, holistic healthcare model that focuses on intervention at the earliest stages of family planning[46,54]. This includes the provision of support with a trauma informed lens, in order to respond to women that are facing complex risk factors associated with exposure to violence, toxic stress and the legacies of colonisation. The implementation of these approaches represents a considerable challenge for policy makers and service providers but are required to alleviate the considerable burden of mental health issues among Aboriginal women at and after birth. 

## Limitations

The estimations in this study can at most be considered an average over the five-year time-span given the possible differences in clinical diagnoses, cultural and demographic factors over those years. Our study is restricted to mental health diagnoses from hospital admissions (HMDC) and contacts with mental health services (MHIS). Accordingly, it does not capture people with undiagnosed conditions and those not receiving assistance for mental health conditions, and likely under-estimates the scale of mental health-related contacts.

The spatial and geographical distribution of the population was measured using the variable Remoteness Areas (RAs) classification developed by ABS. This reflects the actual area of residence of each individual and not necessarily the distribution of health or mental health services.

Given the available data, we were unable to account for relevant proximal determinants of mental health problems (e.g. substance abuse, alcohol and smoking) or more distal, possible upstream contributors to poor mental health (e.g. racism, discrimination and the intergenerational effects of trauma).

## Strengths

One of the key strengths of the study is that it provides a comparative estimate of socioeconomic and geographic remoteness parameters using two mixed effect models, with and without their interaction.

The study draws on total population data in the state of WA, which is large in scale and time period. This includes robust, high-quality data registries that have been linked using internationally accepted best practice procedures.

## Conclusion

This study provides new insights on the dynamic relationship between SES, geographic location and mental health issues among Australian Aboriginal women in the five years leading up to childbirth. We highlight that the pattern of socioeconomic disparities in maternal mental health contacts varies by remoteness of residence, with a reverse gradient observed in urban settings and a traditional pattern evident in more remote areas. The results underscore the need to apply location-specific approaches to addressing the material and psychosocial pathways that lead to mental health problems and the provision of culturally safe, appropriate and accessible services for Aboriginal peoples.

## Ethical Approvals

The study was approved by the Western Australian Aboriginal Health Ethics Committee (reference 416), Murdoch University Human Research Ethics Committee (reference 2014/025) and Western Australian Department of Health WA Human Research Ethics Committee (reference 2014/21). These ethical approvals support a waiver of consent on the basis that the study utilises routinely collected information from existing administrative datasets (and, accordingly, does not include active participants), and only has access to de-identified data, which are stored, analysed and disseminated according to strict protocols.

## Supplementary Material

Supplementary File

## Abbreviations

IRSAD (Index of Relative Advantage/Disadvantage), SEIFA (Socio-Economic Index for Areas), SES (socioeconomic status), AIC (Akaike’s Information Criterion), BIC (Bayesian Information Criterion), LR (Likelihood ratio)
