# Supplementary material for: Psychosocial disadvantage and residential remoteness is associated with Aboriginal women’s mental health prior to childbirth
Source: Int J Popul Data Sci. 2020 Feb 26;5(1):1153. doi: 10.23889/ijpds.v5i1.1153 (PMC7473279; doi:10.23889/ijpds.v5i1.1153)
Supplement: Supplementary File [file ijpds-05-1153-s001.pdf]

Supplementary table 1: Mental Health Related Diagnoses Groups

Mental Health disorders	ICD-9-CM	ICD-10-AM
Organic disorder	290, 293, 294, 310	F00-F09, G30
Substance-related disorder	291, 292, 303, 304, 305	F10-F19, F55
Schizophrenia	295, 296, 297, 298	F20-F29, F33.3, F30, F31, F32.1-F32.8, F33.1-F33.9, F34.8, F34.9, F38, F39
Mood disorder	296, 300.4, 311,	F32.0, F32.9, F33.0, F34.1
Anxiety	300.0, 300.2, 300.3, 300.8, 308, 309	F40-F43, F45,F48, F63.3, F68
Personality disorder	301	F60, F61, F62, F68.1, F68.8, F69
Intellectual disability (IQ)	317, 318, 319	F70-F79
Disorders of psychological development	299, 307.2, 307.3, 307.6, 307.7 312, 313, 314, 315	F80-F89, F90-F98
Intentional self-harm	E950-E959	X6, X7, X80-X84
Other	300.5, 300.6, 300.7, 300.9, 302, 306, 307.0, 307.1, 307.4, 307.5, 307.8, 307.9, 310, 316	F44, F48, F50, F51, F52, F53, F54, F59, F63, F64, F65, F66, F95, F98, F99

Supplementary table 2: Characteristics of the Study Sample and Excluded Mothers

**Total observations (N=45,211)**	**Study Sample (n=39,845)**		**Excluded sample (n=5,366)**	
	**(n)**	**Mean/% (95% CI)**	**(n)**	**Mean/% (95% CI)**
Any mental health contact	**39,845**	27.6 [27.1, 28.0]	**5,366**	28.0 [26.8, 29.2]
Overall mean IRSAD score (deciles)	**39,845**	5.5 [5.5, 5.5]	**1,658**	7.1 [6.9, 7.2]
**REMOTENESS AREAS**	**39,845**		**1,066**	
Metropolitan	(8,916)	22.4 [22.0, 22.8]	(470)	44.1 [41.1, 47.1]
Inner regional	(7,466)	18.7 [18.4, 19.1]	(99)	9.3 [7.6, 11.0]
Outer regional	(3,753)	9.4 [9.1, 9.7]	(149)	14.0 [11.9, 16.1]
Remote	(3,498)	8.8 [8.5, 9.1]	(164)	15.4 [13.2, 17.6]
Very remote	(16,212)	40.7 [40.2, 41.2]	(184)	17.3 [15.0, 19.5]
MARITAL STATUS	**39,845**		**5,330**	
Not married	(13,200)	33.1 [32.7, 33.6]	(3,227)	60.5 [59.2, 61.9]
Widowed	(49)	0.1 [0.09, 0.16]	(3)	0.06 [-0.01, 0.1]
Divorced	(87)	0.2 [0.2, 0.3]	(7)	0.1 [0.03, 0.2]
Separated	(667)	1.7 [1.5, 1.8]	(32)	0.6 [0.4, 0.8]
Married/de facto	(25,420)	63.8 [63.3, 64.3]	(1,959)	36.7 [35.5, 38.0]
Not stated	(422)	1.1 [1.0, 1.2]	(102)	1.9 [1.5, 2.3]
Maternal age	**39,845**	24.7 [24.6, 24.7]	**5,330**	25.1 [24.9, 25.3]
Mothers with 1 or more children	**39,845**	71.0 [63.1, 64.6]	**3,940**	79.5 [31.7, 35.2]

Supplementary table 3. Akaike's Information Criterion and Bayesian Information Criterion comparison between fixed effects and mixed effects logistic regression models

	Obs	LL (null)	LL (model)	DF	AIC	BIC
Table 2- Model 1	39845	-23455.55	-23369.63	6	46751.27	46802.82
Table 2- Model 2	39845	-23455.55	-23308.14	11	46638.27	46732.79
Table 2- Model 3	39845	-23455.55	-23064.72	13	46155.45	46267.16
**Nested Models**						
Table 2- Model 4 (no interaction)	39845	not applicable	-23008.56	14	46045.11	46165.41
Table 3. (with interaction between IRSAD decile & Remoteness)	39845	not applicable.	-22982.76	18	46001.52	46001.52

Note: LL – log likelihood; df – degrees of freedom;

AIC - Akaike's Information criterion;

BIC - Bayesian information criterion;

N=Obs used in calculating BIC

Supplementary table 4. Pairwise Comparisons of Predictions, Without Adjustment for Multiple Comparison

	Contrast	Std. Err.	Z	P>|z|
Inner regional vs Metropolitan	-0.011	.02	-0.54	0.588
Outer regional vs Metropolitan	-0.082	.02	-3.71	0.000
Remote vs Metropolitan	-0.064	.02	-2.87	0.004
Very Remote vs Metropolitan	-0.097	.02	-5.77	0.000
Outer regional vs Inner-regional	-0.071	.02	-3.28	0.001
Remote vs Inner regional	-0.053	.02	-2.69	0.016
Very remote vs Inner-regional	-0.086	.02	-5.30	0.000
Remote vs Outer regional	0.019	.02	0.79	0.428
Very Remote vs Outer-regional	-0.014	.02	-0.79	0.432
Very Remote vs Remote	-0.033	.02	-1.87	0.061

Supplementary table 5. Pairwise Comparisons of Predictions, With Adjustment for Multiple Comparison

	Contrast	Std. Err.	Z	P>|z|
Inner regional vs Metropolitan	-0.011	.02	-0.54	0.990
Outer regional vs Metropolitan	-0.082	.02	-3.71	0.008
Remote vs Metropolitan	-0.064	.02	-2.87	0.083
Very remote vs Metropolitan	-0.097	.02	-5.77	0.000
Outer regional vs Inner-regional	-0.071	.02	-3.28	0.029
Remote vs Inner regional	-0.053	.02	-2.42	0.210
Very remote vs Inner-regional	-0.086	.02	-5.30	0.000
Remote vs Outer regional	0.019	.02	0.79	0.960
Very remote vs Outer-regional	-0.014	.02	-0.79	0.961
Very remote vs Remote	-0.033	.02	-1.87	0.477

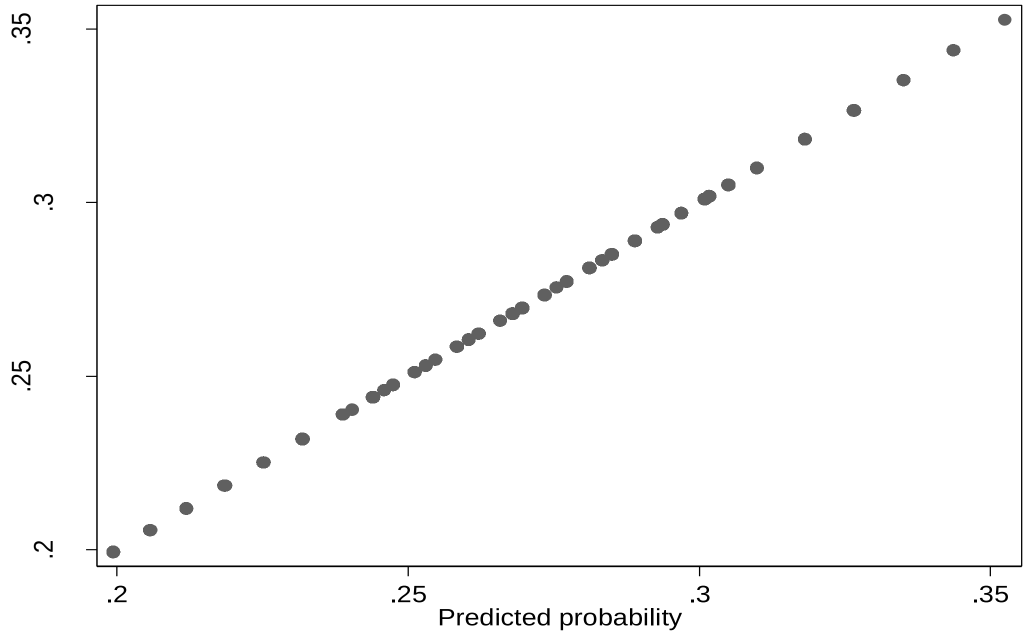

Supplementary figure 1. Change in Pearson chi-squared by Predicted Margins (Probability) of Mental Health Contact

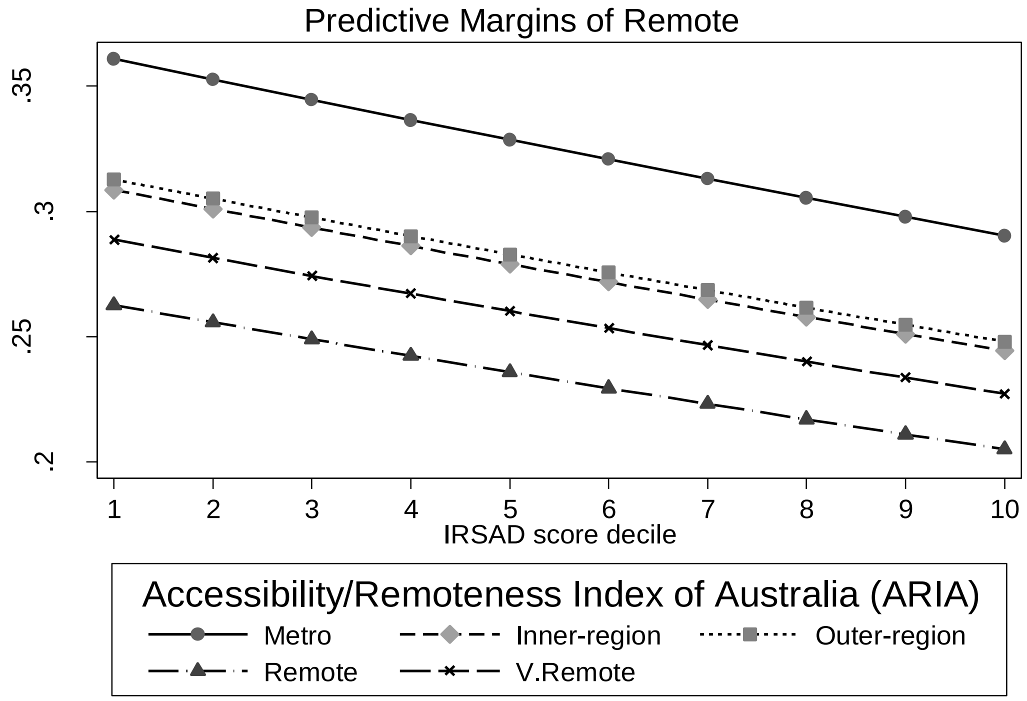

Supplementary figure 2. Predicted Margins (Probability) of Mental Health Contact by IRSAD-decile-score and Remoteness, from Saturated Model with No Interaction-term

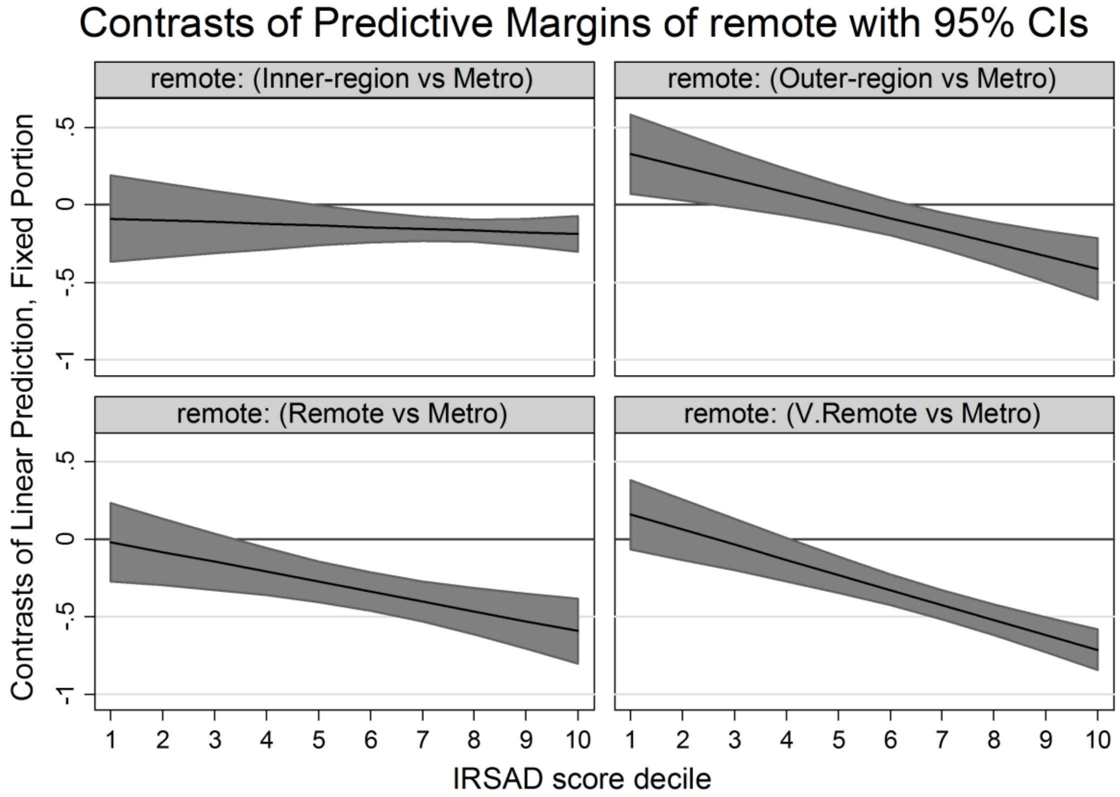

Supplementary figure 3. Mental Health Contact Probability Contrasts by IRSAD-decile and Remoteness-level

IRSAD = Index of Relative Socioeconomic Advantage and Disadvantage
